# Supplementary material for: Adenomyosis in women undergoing hysterectomy for abnormal uterine bleeding associated with uterine leiomyomas
Source: PLoS One. 2023 Dec 11;18(12):e0294925. doi: 10.1371/journal.pone.0294925 (PMC10712893; doi:10.1371/journal.pone.0294925)
Supplement: Attachment — Submitted filename: NLonky Response to Reviewers Memo_2023Apr05.docx [file pone.0294925.s001.docx]

**Global suggestions:**

1. *Please complete all items on the Clinical Studies Checklist*

**Response:** We have submitted the Clinical Studies Checklist in the resubmission of the manuscript. We indicated on the Checklist the line numbers where clarification was made in the Methods section of the manuscript.

*2. Please upload a clean copy of your manuscript that does not contain any track changes. Upload this as your “Manuscript” file.*

**Response:** We have provided a clean copy of the manuscript, without track changes.

*3. Please upload a new copy of Figure 1 as the detail is not clear.*

**Response:** We have updated the Figure 1 with 600 dpi to improve the resolution.

*4. Please provide additional details regarding participant consent. In the ethics statement in the Methods and online submission information, please ensure that you have specified (1) whether consent was informed and (2) what type you obtained (for instance, written or verbal, and if verbal, how it was documented and witnessed). If you are reporting a* ***retrospective study*** *of medical records or archived samples, please ensure that you have discussed whether all data were fully anonymized before you accessed them and/or whether the IRB or ethics committee waived the requirement for informed consent. If patients provided informed written consent to have data from their medical records used in research, please include this information.*

**Response:** In this retrospective data only study, the research involved no more than

minimal risk to participants and involved no procedures for which signed consent is normally

required thus we recived a waiver of written informed consent from the KPSC Institutional

Review Board. All study staff had access to the medical records of the identified cohort from

the database, for chart abstraction and review of radiology reports. However, during analysis,

the data set was aggregated and fully anonymized. This information was included in lines

156-158 of the manuscript.
