# Supplementary material for: Adenomyosis in women undergoing hysterectomy for abnormal uterine bleeding associated with uterine leiomyomas
Source: PLoS One. 2023 Dec 11;18(12):e0294925. doi: 10.1371/journal.pone.0294925 (PMC10712893; doi:10.1371/journal.pone.0294925)
Supplement: Attachment — Submitted filename: Response to Reviewers.docx [file pone.0294925.s002.docx]

Dear Editor,

We thank you and the reviewer for the positive feedback and the constructive comments provided. Our responses to reviewer comments are listed below and the changes made in the manuscript are tracked with tracked change. We appreciate the opportunity to revise and resubmit our manuscript and look forward to hearing back from **PLOS ONE.**

**Editor’s Specific Comments**:

***When submitting your revision, we need you to address these additional requirements.***

***1. Please ensure that your manuscript meets PLOS ONE's style requirements, including those for file naming. The PLOS ONE style templates can be found at***

[***https://journals.plos.org/plosone/s/file?id=wjVg/PLOSOne_formatting_sample_main_body.pdf***](https://urldefense.com/v3/__https:/journals.plos.org/plosone/s/file?id=wjVg*PLOSOne_formatting_sample_main_body.pdf__;Lw!!BZ50a36bapWJ!qjf7wbo8lmyuDY3EYejCKUNIAQMaXJDKWtGLoG6SlBov4z9mxLU6h_7is9D0G__HpWIT65U50bFZEUfV6Lxn$) ***and***

[***https://journals.plos.org/plosone/s/file?id=ba62/PLOSOne_formatting_sample_title_authors_affiliations.pdf***](https://urldefense.com/v3/__https:/journals.plos.org/plosone/s/file?id=ba62*PLOSOne_formatting_sample_title_authors_affiliations.pdf__;Lw!!BZ50a36bapWJ!qjf7wbo8lmyuDY3EYejCKUNIAQMaXJDKWtGLoG6SlBov4z9mxLU6h_7is9D0G__HpWIT65U50bFZEc7UhVEo$)

**Response:** We have addressed any style requirements, including file naming using the PLOS ONE style templates.

***2. Thank you for stating the following financial disclosure:***

***"Dr. Neal Lonky, Principal Investigator received funding from AbbVie Inc. The funders provided feedback on the scope of work protocol. Contract #00115992.0"***

***Please state what role the funders took in the study.  If the funders had no role, please state: "The funders had no role in study design, data collection and analysis, decision to publish, or preparation of the manuscript."***

***If this statement is not correct you must amend it as needed.***

***Please include this amended Role of Funder statement in your cover letter; we will change the online submission form on your behalf.***

**Response:** The funders had no role in study design, data collection and analysis, decision to publish, or preparation of the manuscript.

***3. Thank you for stating the following in the Competing Interests/Financial Disclosure * (delete as necessary) section:***

***"This manuscript is original research and the authors of this manuscript, and financial disclosures include the following:***

***o             Dr. Lonky reports a consultancy agreement with AbbVie following conclusion of this research, research support from AbbVie and Merk & Co. is and is the founder/shareholder of Histologics LLC.***

***o             Dr Munro reports consultancy agreements with AbbVie Inc, American Regent, Daiichi Sankyo, Hologic Inc, Myovant Inc, Pharmacosmos, and Vifor Pharmaceuticals. He has also received indirect grant support from AbbVie Inc and Pharmacosmos."***

***We note that you received funding from a commercial source:"AbbVie and Merk & Co, American Regent, Daiichi Sankyo, Hologic Inc, Myovant Inc, Pharmacosmos, and Vifor Pharmaceuticals"***

***Please provide an amended Competing Interests Statement that explicitly states this commercial funder, along with any other relevant declarations relating to employment, consultancy, patents, products in development, marketed products, etc.***

***Within this Competing Interests Statement, please confirm that this does not alter your adherence to all PLOS ONE policies on sharing data and materials by including the following statement: "This does not alter our adherence to PLOS ONE policies on sharing data and materials.” (as detailed online in our guide for authors*** [***http://journals.plos.org/plosone/s/competing-interests***](https://urldefense.com/v3/__http:/journals.plos.org/plosone/s/competing-interests__;!!BZ50a36bapWJ!qjf7wbo8lmyuDY3EYejCKUNIAQMaXJDKWtGLoG6SlBov4z9mxLU6h_7is9D0G__HpWIT65U50bFZEXn5nJGV$)***).  If there are restrictions on sharing of data and/or materials, please state these. Please note that we cannot proceed with consideration of your article until this information has been declared.***

***Please include your amended Competing Interests Statement within your cover letter. We will change the online submission form on your behalf.***

**Response:** Please see the amended Competing Interests Statement below:

Dr. Lonky reports a consultancy agreement with AbbVie following conclusion of this research, research support from AbbVie and Merk & Co. is the founder/shareholder of Histologics LLC.Dr Munro reports consultancy agreements with AbbVie Inc, American Regent, Daiichi Sankyo, Hologic Inc, Myovant Inc, Pharmacosmos, and Vifor Pharmaceuticals. He has also received indirect grant support from AbbVie Inc and Pharmacosmos. This does not alter our adherence to PLOS ONE policies on sharing data and materials.

***4. Please amend the manuscript submission data (via Edit Submission) to include author "Malcolm G. Munro"***

**Response:** We amended the manuscript submission data to include author “Malcolm G. Munro.”

***5. In your Data Availability statement, you have not specified where the minimal data set underlying the results described in your manuscript can be found. PLOS defines a study's minimal data set as the underlying data used to reach the conclusions drawn in the manuscript and any additional data required to replicate the reported study findings in their entirety. All PLOS journals require that the minimal data set be made fully available. For more information about our data policy, please see*** [***http://journals.plos.org/plosone/s/data-availability***](https://urldefense.com/v3/__http:/journals.plos.org/plosone/s/data-availability__;!!BZ50a36bapWJ!qjf7wbo8lmyuDY3EYejCKUNIAQMaXJDKWtGLoG6SlBov4z9mxLU6h_7is9D0G__HpWIT65U50bFZEc9t3thA$)***.***

***Upon re-submitting your revised manuscript, please upload your study’s minimal underlying data set as either Supporting Information files or to a stable, public repository and include the relevant URLs, DOIs, or accession numbers within your revised cover letter. For a list of acceptable repositories, please see*** [***http://journals.plos.org/plosone/s/data-availability#loc-recommended-repositories***](https://urldefense.com/v3/__http:/journals.plos.org/plosone/s/data-availability*loc-recommended-repositories__;Iw!!BZ50a36bapWJ!qjf7wbo8lmyuDY3EYejCKUNIAQMaXJDKWtGLoG6SlBov4z9mxLU6h_7is9D0G__HpWIT65U50bFZEXMTPJMn$)***. Any potentially identifying patient information must be fully anonymized.***

***Important: If there are ethical or legal restrictions to sharing your data publicly, please explain these restrictions in detail. Please see our guidelines for more information on what we consider unacceptable restrictions to publicly sharing data:*** [***http://journals.plos.org/plosone/s/data-availability#loc-unacceptable-data-access-restrictions***](https://urldefense.com/v3/__http:/journals.plos.org/plosone/s/data-availability*loc-unacceptable-data-access-restrictions__;Iw!!BZ50a36bapWJ!qjf7wbo8lmyuDY3EYejCKUNIAQMaXJDKWtGLoG6SlBov4z9mxLU6h_7is9D0G__HpWIT65U50bFZEVSWPCFJ$)***. Note that it is not acceptable for the authors to be the sole named individuals responsible for ensuring data access.***

***We will update your Data Availability statement to reflect the information you provide in your cover letter***

**Response:** Please see our Data Availability statement below:

Individual-level data reported in this study involving human research participants are not publicly shared due to potentially identifying or sensitive patient information. Upon request, and subject to review, the institutions may provide deidentified aggregate-level data that support the findings of this study. Anonymized data (deidentified data including participant data as applicable) that support the findings of this study may be made available from the investigative team in the following conditions: 1) agreement to collaborate with the study team on all publications, (2) provision of external funding for administrative and investigator time necessary for this collaboration, (3) demonstration that the external investigative team is qualified and has documented evidence of training for human subjects protections, and (4) agreement to abide by the terms outlined in data use agreements between institutions. Interested researchers should contact Kaiser Permanente Southern California of Research & Evaluation Compliance Assurance Services at: ResEvalCAS@kp.org.

**Reviewer #1:**

***Thank you to giving me the chance to review this paper.***

***despite good overall merit, I have some comments:***

***-please discuss on relevant literature on the prevalence of adenomyosis in women with endometriosis, comparing your data with those from these letters (eg doi 10.1016/j.ejogrb.2014.08.016).***

**Response**: We have added our data on the prevalence of endometriosis in our cohort, as well as appropriate citations. We do emphasize that our study design was such that histopathological diagnosis was minimally contributory while we had to rely upon operative reports to determine whether or not endometriosis was present at the time of surgery. Because there was no structured method for entering these data, the prevalence of endometriosis in this cohort is likely underreported.

***- US diagnosis of adenomyosis is difficult also for expert operators, many efforts should be done to optimize its diagnostic performance and gain standardization of diagnostic criteria; discuss on novel findings on these topics (eg doi 10.3390/jpm12101572; PMID: 36767092***

**Response:** We thank the reviewer for this suggestion but feel that discussion of the standardization of diagnostic criteria is beyond the scope of our work. In this study we asked an experienced radiologist to retrospectively evaluate ultrasounds for the presence or absence of adenomyosis but did not evaluate by criteria. We have dealt with our findings in the context of what is known, particularly about the sensitivity of ultrasound for adenomyosis in the presence of uterine leiomyomas. starting in lines 397-419.
